# Investigation of SARS-CoV-2 inactivation using UV-C LEDs in public environments via ray-tracing simulation

**DOI:** 10.1038/s41598-021-02156-8

**Published:** 2021-11-19

**Authors:** Po-Yen Lai, Huizhe Liu, Ray Jia Hong Ng, Bianca Wint Hnin Thet, Hong-Son Chu, Jin Wah Ronnie Teo, Qunxiang Ong, Yuanjie Liu, Ching Eng Png

**Affiliations:** 1 A*STAR Institute of High Performance Computing, Electronics and Photonics, 1 Fusionopolis Way, #16-16, Connexis, 138632 Singapore; 2 A*STAR Singapore Institute of Manufacturing Technology, 2 Fusionopolis Way, #08-04, Innovis, 138634 Singapore; 3 A*STAR Singapore Bioimaging Consortium, 11 Biopolis Way, #02-02, Helios, 138667 Singapore; 4 A*STAR National Metrology Centre, 2 Fusionopolis Way, #08-05, Innovis, 138634 Singapore

**Keywords:** Optics and photonics, Disease prevention, Pathogens

## Abstract

This paper proposes an investigating SARS-CoV-2 inactivation on surfaces with UV-C LED irradiation using our in-house-developed ray-tracing simulator. The results are benchmarked with experiments and Zemax OpticStudio commercial software simulation to demonstrate our simulator's easy accessibility and high reliability. The tool can input the radiant profile of the flexible LED source and accurately yield the irradiance distribution emitted from an LED-based system in 3D environments. The UV-C operating space can be divided into the safe, buffer, and germicidal zones for setting up a UV-C LED system. Based on the published measurement data, the level of SARS-CoV-2 inactivation has been defined as a function of UV-C irradiation. A realistic case of public space, i.e., a food court in Singapore, has been numerically investigated to demonstrate the relative impact of environmental UV-C attenuation on the SARS-CoV-2 inactivation. We optimise a specific UV-C LED germicidal system and its corresponding exposure time according to the simulation results. These ray-tracing-based simulations provide a useful guideline for safe deployment and efficient design for germicidal UV-C LED technology.

## Introduction

Coronavirus disease 2019 (COVID-19), caused by severe acute respiratory syndrome coronavirus 2 (SARS-CoV-2), was first detected in December 2019 in Wuhan, China, and has since sparked a global pandemic. A respiratory infection such as SARS-CoV-2 can be transmitted from direct physical contact with the virus from an infected person’s respiratory fluids or via droplets on surfaces and aerosol transmission^1,2^. As a consequence, internationally coordinated efforts have been put into large-scale medical, scientific, economic, and public undertakings to prevent fomite transmission, particularly for high touch surfaces in public environments^3^.

Existing surface disinfection approaches such as chemical spraying^4^, thermal treatment^5^, or surface wiping can be labor-intensive, costly, and consumes high energy. Therefore, it is imperative to develop a safe and effective non-contact sanitising solution to prevent fomite transmission in public spaces. Light-based inactivation using Ultraviolet-C (UV-C) light sources (220 < λ <280 nm) has been proven to be one of the most efficient ways of inactivating a wide range of microbes and viruses by destroying their DNAs or RNAs, including SARS-CoV-2^6,7^. While germicidal UV-C Mercury (Hg) lamp is widely used in water treatment and air handling units^8^, the directionality of LED offers a more targeted and safer alternative compared to the Hg lamp. It also exhibits several advantageous features such as environmental friendliness, energy savings, compact size, low cost, and durability^9^. The inactivation of SARS-CoV-2 using UV-C LED also becomes a significant concern for its practical importance in mitigating COVID-19 transmission. The works^10,11^ performed the study for the reduction of airborne SARS-CoV-2 spread using far-UV-C (λ = 222 nm) LEDs. Similarly, several articles^12–14^ showed that the LEDs within a UV-C range (265 < λ < 280 nm) are the promising light source to inactivate SARS-CoV-2 by photo-degradation of spike protein rapidly. Moreover, Liu et al.^15^ proposed the AlGaN-based LEDs (λ = 275 nm) structure design and epitaxy optimisation for achieving a fast inactivation of SARS-CoV-2 using high-power UV-C irradiation. However, one major drawback of UV-C germicidal irradiation is its harmful effect on human eyes and skin, so that there are international safety guidelines to limit human exposure^16,17^.

Optical simulations can readily guide not only the efficient design of germicidal UV-C LED but implementation of safe deployment which is particularly important as UV-C wavelength is not visible to naked human eyes. Currently, there are only a few numerical systematic studies^10,10^ on UV-C radiation mapping in public environments. The work^10^ proposed a coupled-CFD radiative model for predicting airborne SARS-CoV-2 inactivation in a single-occupancy private room in hospitals, a simplified 2D system in simulations. Another recent work by Hou et al.^18^ conducted a parametric study of UV-C germicidal technology in a patient room in hospitals by adjusting the room configuration, ceiling height, and surface materials using ray-tracing simulation. Apart from the proposed numerical works^10,10^ for evaluating the UV-C germicidal effectiveness in medical facilities, the simulation demonstrations on three-dimensional (3D) UV-C mapping in environments of day-to-day life are still lacking. In addition, the systematic study of both UV-C LEDs in inactivation on surfaces of the complex 3D environments and the margin of safety for UV-C LEDs are indispensable during the COVID-19 pandemic.

In this work, we develop an in-house ray-tracing (RT) simulator based on UV-C LEDs to inactivate SARS-CoV-2 in public environments. Based on a physically accurate and geometrically flexible RT, our proposed simulator can model the specific design of a UV-C LED array and generate the corresponding irradiance map in a large-scale ambient operating environment. The simulated irradiance map guides a defined safe boundary with a designated inactivation zone during the germicidal operation. Furthermore, these RT simulations help optimise the light source's distance and determine the exposure time for a sufficient UV-C dose. In addition, environmental UV-C attenuations^7,19^ due to superficial dirt and substances in the air have been summarized as compact mathematical forms and considered in the RT simulator. The methodology of this work, including the RT model, the experimental setup for benchmark, and the quantitative determination of SARS-CoV-2 inactivation, is described in “Methods” section. The case study of a public environment based on 3D RT simulations and the parametric analysis considering ambient environmental attenuations is presented in the following section. Finally, the discussion, concluding remarks, and outlook on the work are given.

## Methods

### UV-C LED radiation model

**Figure 1:**
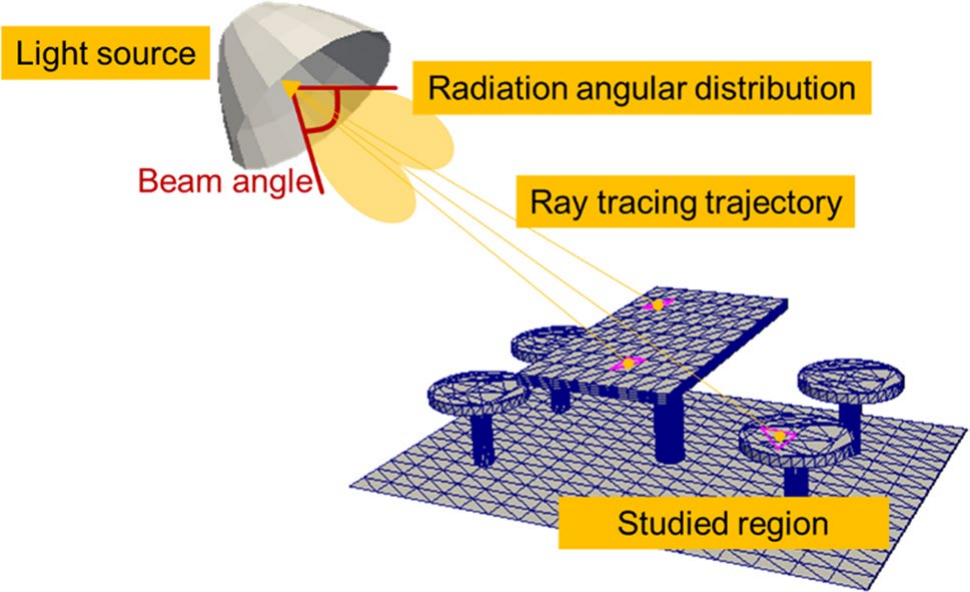
Schematic illustration of the RT model to predict the radiation level in the area of interest.

In this section, we proposed an RT model to predict the UV-C radiation level in the area of interest. The model is developed based on the RT technique that is physically accurate, highly flexible with geometrical input, and easy to parallelize. In Fig. 1, the light source is defined by its structure (e.g., single LED or LED array) and amplitude spatial distribution, including the beam angle and radiation angular distribution. Next, the complex studied region is geometrically described in STereoLithography (STL) format, in which uniform triangle meshes are automatically generated for a suitable mesh resolution. Finally, the backward RT technique is applied to trace the rays from each mesh point to the light source.

The irradiance $\phi$ [W/m^2^] in the far-field can be expressed as a function of radiant intensity $I\left(\theta \right)$ [W/sr], in which $\theta$ is a polar angle in a coordinate system centered in the emission source^20^, given by

1 $$\phi = \frac{{I(\theta )cos\theta_{d} }}{{r^{2} }},$$

where ${\theta }_{d}$ the incident angle to the detector mesh point, and *r* is the distance between the source and the detector. The total irradiance on a detector from a given UV-C LED module consists of *N* point sources (i.e., LEDs) at an operating wavelength ${\lambda }_{0}$ with total power ${P}_{s, {\lambda }_{0}}$ can be re-written as

2 $$\phi_{{d,\lambda_{0} }} = \frac{{P_{{s,\lambda_{0} }} /N}}{{2\pi \int_{ - \pi /2}^{\pi /2} {I_{{n,\lambda_{0} }} (\theta_{s} )\sin \theta_{s}d\theta_{s} } }}\sum\nolimits_{N} {\frac{{I_{{n,\lambda_{0} }} (\theta_{s} )\cos \theta_{d} }}{{(x_{s} - x_{d} )^{2} + (y_{s} - y_{d} )^{2} + (z_{s} - z_{d} )^{2} }}} ,$$

where ${I}_{n,{\lambda }_{0}}\left({\theta }_{s}\right)$ is the normalized radiant intensity, also called the angular radiant distribution, and ${\theta }_{s}$ is the polar angle in a coordinate system centered in the point source. The coordinates (*x*_*s*_, *y*_*s*_, *z*_*s*_) and (*x*_*d*_, *y*_*d*_, *z*_*d*_) are the light source positions and the detector mesh point, respectively. From Eq. (2), the information about the distances between the LEDs in the LED module is included in the summation of all the sources, as the different LED sources will have different spatial coordinates (*x*_*s*_, *y*_*s*_, *z*_*s*_), as well as different angles *θ*_s_ and *θ*_d_, giving different values for the integrand in the calculation of the irradiance for the respective sources.

**Figure 2:**
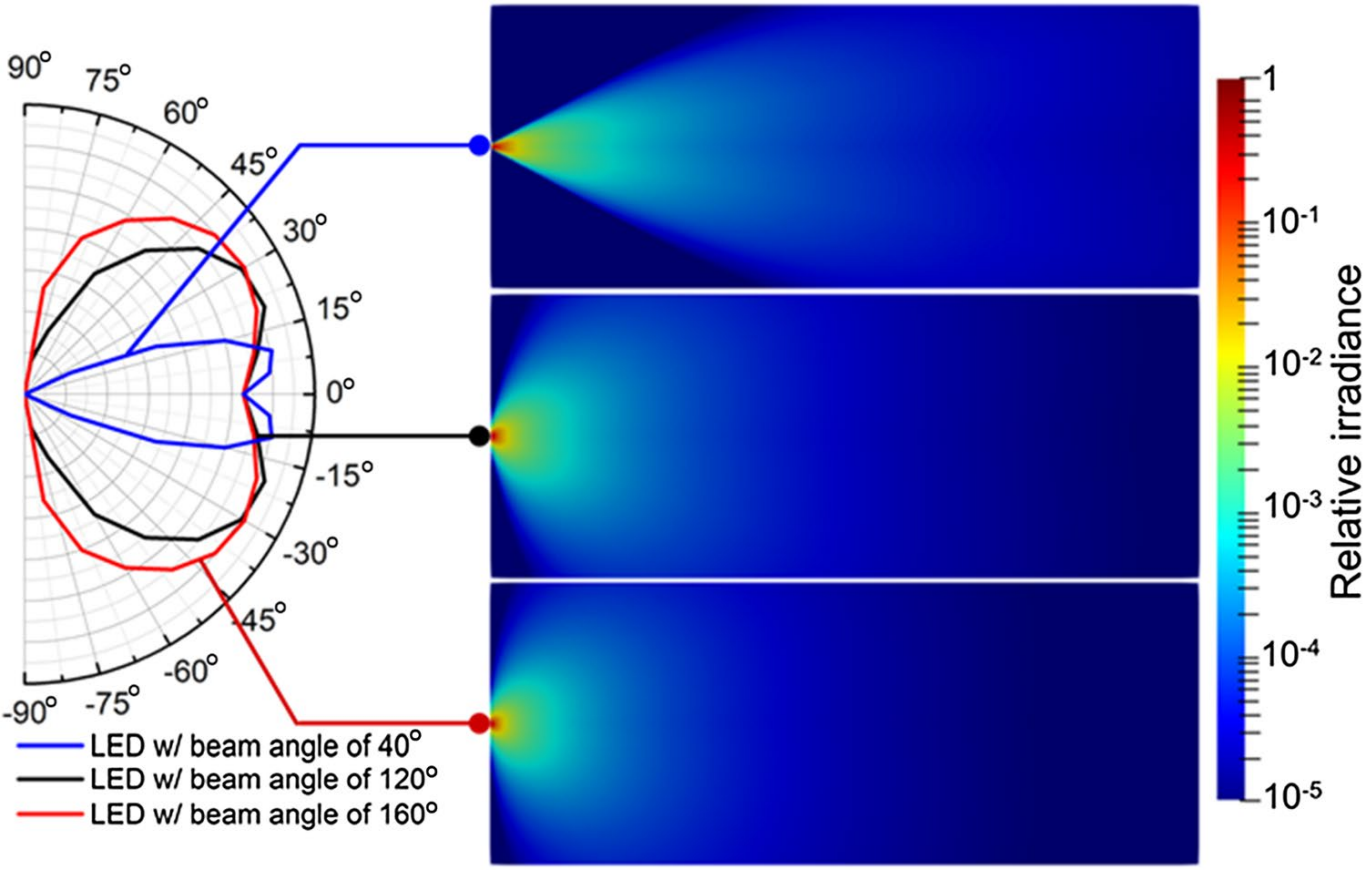
Radiation angular profile and irradiance of UV LED with the beam angle of 40°, 120°, and 160°, respectively.

Figure 2 shows the irradiance distributions on 2D cut-planes predicted by the RT model where the light sources are the single LED with a beam angle of 40°, 120°, and 160°, respectively. In the left panel of Fig. 2, these Lambertian-like radiation profiles^20^ are the common types of UV-C LED for sterilization^21^ which gives the angular radiant distribution ${I}_{n,{\lambda }_{0}}\left({\theta }_{s}\right)$ in Eq. (2). The corresponding cut-plane irradiance distribution, calculated from Eq. (2), varies as the beam angle is changed. Each LED is assumed as a point source with a given radiation profile based on the far-field approximation in this work. In this case, it is convenient to demonstrate the design of an LED array using the linear superposition of the irradiance from different single LEDs.

### Model validation

**Figure 3:**
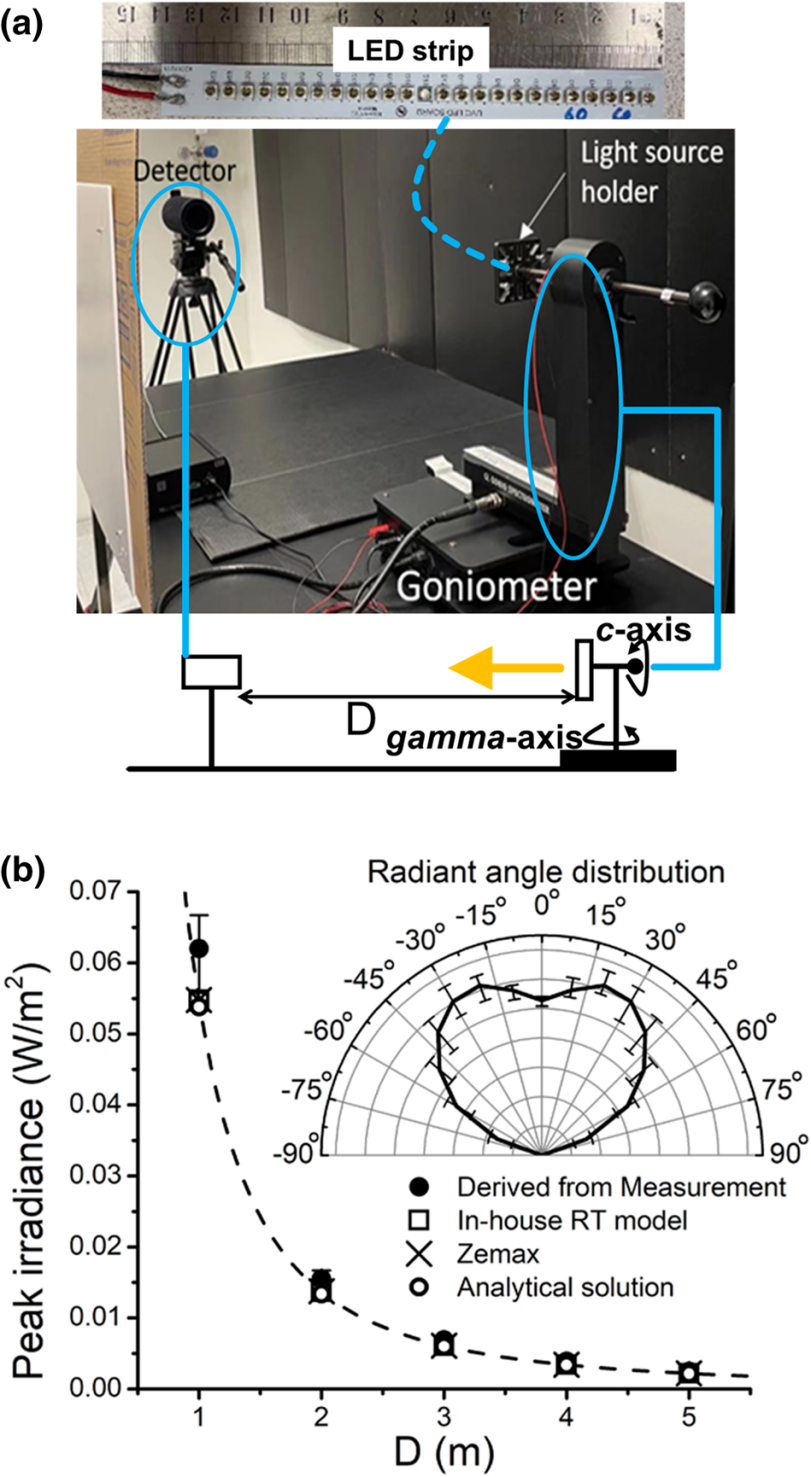
(**a**) The picture of the experimental setup and the schematic illustration for measuring the radiation emitted by LED strips are shown. (**b**) The model validation by comparing the peak irradiance derived from the measurement and the simulation done by the in-house-developed RT model, Zemax OpticStudio, and the analytical solution. The inset in (**b**) shows the angular radiation pattern of a single LED. The error bars comes from the standard deviation of the average in *c* angles.

Figure 3 shows the experimental setup for measuring the radiation profile of the UV-C LED light source. The upper picture in Fig. 3a shows the UV-C LED strip light source consisting of a linear arrangement of 25 single LEDs spaced 0.005 m apart with a measured total power of 0.194 W and a peak wavelength of ~ 274 nm. The middle picture in Fig. 3a presents the measurement setup of the UV-C light source. The light source under test is attached to a rotatable holder of a goniometer to measure the spatial light distribution at a distance of 1.5 m apart. The UV-C irradiation measurement setup is calibrated and validated using standards traceable to The International System of Units (SI). As shown in the schematic illustration at the bottom of Fig. 3a, the light source-under-test can be rotated around the *c*-axis and gamma-axis of the spectrometer, and the irradiance at the detector is recorded. The *c*-axis is perpendicular to the LED strip’s luminous surface, and the light source is rotated at 15° intervals between 0° ≤ *c* ≤ 345°. At each *c* angle, the light source is rotated about the gamma (*γ*) axis parallel to the light source's luminous surface under test at 10° intervals between 0° ≤ *γ* ≤ 70°. To validate the RT model, Fig. 3b shows the model predictions of peak irradiance at different distances to the light source and compares it to the experimental measurement. There is a good agreement between them. For the sake of simplicity, the detected radiant profile is averaged out over the *c*-axis, and this averaged angular distribution (see the inset in Fig. 3b) is defined as the input angular radiation profile for the LED light source. Figure 3b also shows the comparison between results simulated using the in-house-developed RT solver and the commercial software, Zemax OpticStudio^22^. They are well agreed for a given input angular radiation profile.

### Inactivation coefficient of SARS‑CoV‑2

While SARS‑CoV‑2 is exposed to UV-C light, the viral concentration decays exponentially as a function of time and the UV-C dosage. The experimental data are well described by the relation^12^:

3 $$S = 1 - e^{ - KD} ,$$

where *S* and *D* represent the sterilizing rate of SARS‑CoV‑2 and the UV-C dose (in the unit of *J*/*m*^2^) at the target surface.

**Figure 4:**
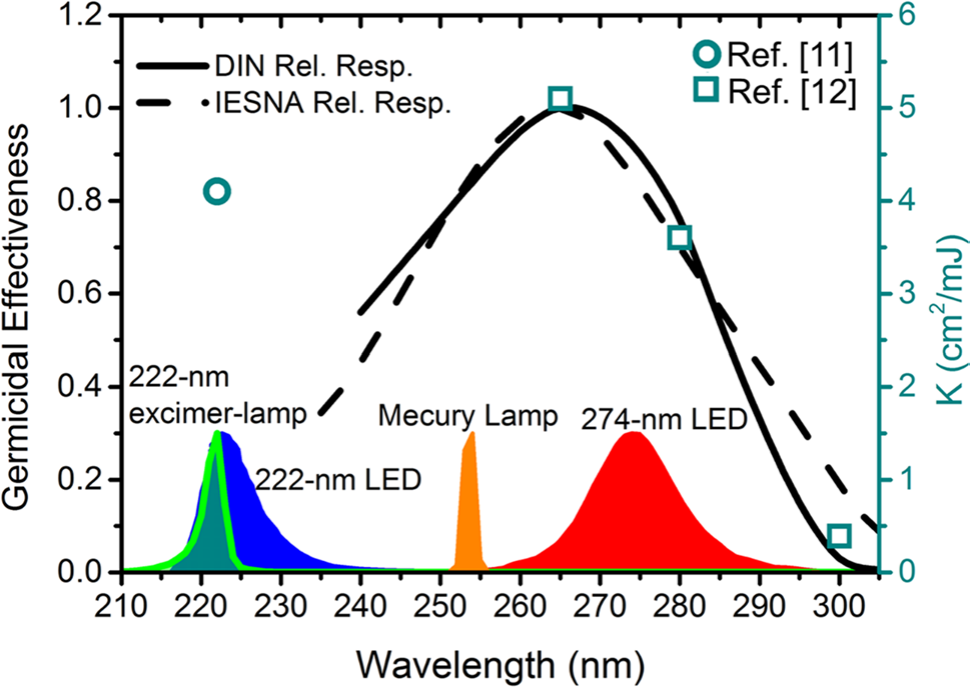
The germicidal effectiveness provided by Deutsches Institut für Normung (DIN)^23^ and Illuminating Engineering Society of North America (IESNA)^24^, and SARS-CoV-2 inactivation coefficient *K* versus radiation wavelength in which the scatter points achieved from the experiments^11^,^12^. The typical spectra of excimer lamp (luminous green line), UV-C LEDs (blue for 222-nm and red for 274-nm), and mercury lamp (orange) are shown at the bottom of the figure.

*K* is a factor called the inactivation coefficient that can be estimated from the survivorship curves by fitting the experimental data^12^. Figure 4 shows *K*^11,12^ as a function of exposure wavelength at 222 nm, 265 nm, 280 nm, and 300 nm, respectively. The trend of *K* is close to the germicidal effectiveness provided by Deutsches Institut für Normung (DIN)^23^ and Illuminating Engineering Society of North America (IESNA)^24^. To evaluate the effectiveness of SARS‑CoV‑2 inactivation on the operating wavelength at 274 nm, we suppose the corresponding value of *K* using linear interpolation based on the experimental data^11,12^. *K* values at 222 nm and 274 nm are close to 4.1 where the *K* value for 222 nm is directly obtained from the experiment^11^ and the *K* value for 274 nm is estimated from the linear interpolation.

By considering the radiative attenuation effect in practical environments, the general expression of the UV-C dose *D* at distance *r* from the light source during the exposure time $\tau$ can be expressed as

4 $$D = \left( {1 - \eta } \right)e^{ - \alpha r} \phi (r)\tau ,$$

where $\phi (r)$, *η*, and *α* respectively represent the UV-C intensity at the target surface, the attenuation factor due to the surface moisture and dirt, and the extinction coefficient due to the radiative loss in a medium. $\phi (r)$ can be numerically calculated using the RT model. For the sake of simplicity, *η* and *α* are assumed as constants of *r* and $\tau$ that are given values for certain conditions.

## Results

In this section, we perform a numerical study for the UV-C sterilization dosage using a specific-designed LED germicidal system in a typical Singapore food court. It is one of the important common public spaces in Singapore, which is an integral part of the way of life for Singaporeans, where people from all walks of life gather at food court centres to dine and bond over their favourite food. In the following simulations, open boundary conditions are adopted excepted for the ground because the distances from the area of concern to the walls and ceil are > 5 times of *R*_eff_. The UV-C reflections from the surface of the food court are neglected due to of commonly found materials (e.g., PVC^25^ and ceramic tiles^26^) in a food court and a doubled optical path (i.e., the inverse-square law of light).

### Case study of a typical food court in Singapore

**Figure 5:**
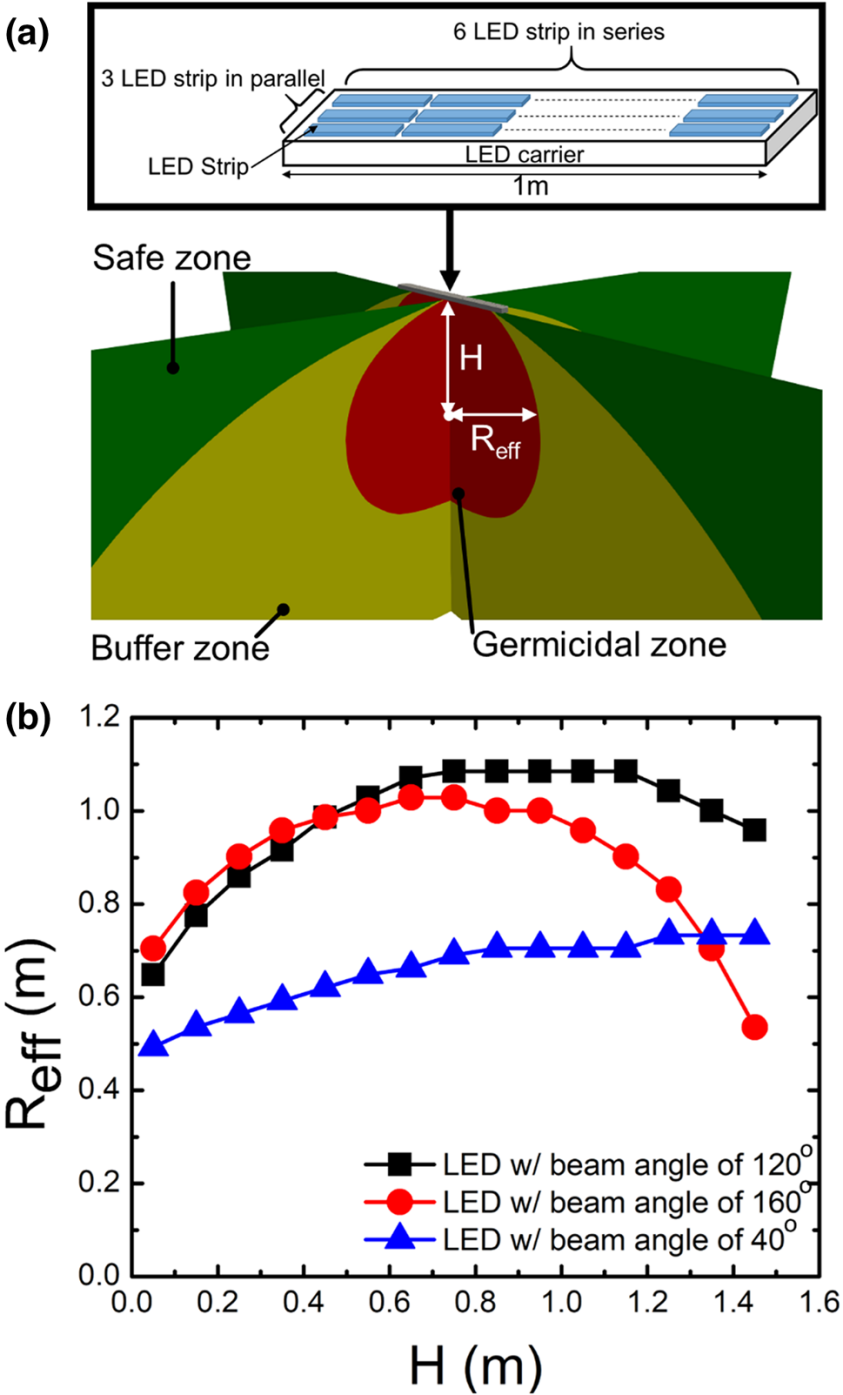
(**a**) The germicidal system designed with LED array composed of 3 × 6 LED strips is shown in the upper panel. The lower panel shows the vertical slice views of irradiance pattern emitted from the LED array in which the space can be divided into the safe zone in green (i.e., < 0.1μW/cm^2^), the buffer zone in yellow, and the effective zone in red (i.e., > 18.7μW/cm^2^). *R*_eff_ is the radius for the effective germicidal region, for a particular distance *H* between the LED array and the detection point. (**b**) The correlation between *H* and *R*_eff_ at the beam angle of 120°, 160°, and 40°.

The configuration of a germicidal system designed with an array of 3 × 6 UV LED strips is illustrated in Fig. 5a. The UV-C LED germicidal system can be handheld or mounted onto a mobile robot. In the upper panel of Fig. 5a, the UV LED strips are embedded in the LED carrier. Two vertical slices of the calculated radiation pattern emitted from the LED array are shown in the lower panel of Fig. 5a. Depending on the emitted irradiance level, the exposure space is divided into the effective germicidal zone with emitted irradiance of > 18.7 μW/cm^2^ for which a 90% inactivation of SARS-CoV-2 can be achieved within half a minute, the safe zone with emitted irradiance of < 0.1 μW/cm^2^ according to international guidelines^16,17,27^, and the buffer zone in between. For the effective germicidal purpose, Fig. 5b shows the radius (i.e., *R*_eff_) of the zone along with the distance (i.e., *H*) from the center of the LED array under LEDs with different beam angles. *R*_eff_ increases with *H* increased until *R*_eff_ reaches the maximum value. *R*_eff_ is proportional to the beam angle of the composed LEDs for the small values of *H*. However, the array composed of LEDs with a beam angle of 120° exhibits the largest *R*_eff_ among the three cases due to the specific design of the array, as shown in Fig. 5a. Moreover, the volume of the germicidal zone reaches the maximum while *H* is around 1.1.

**Figure 6:**
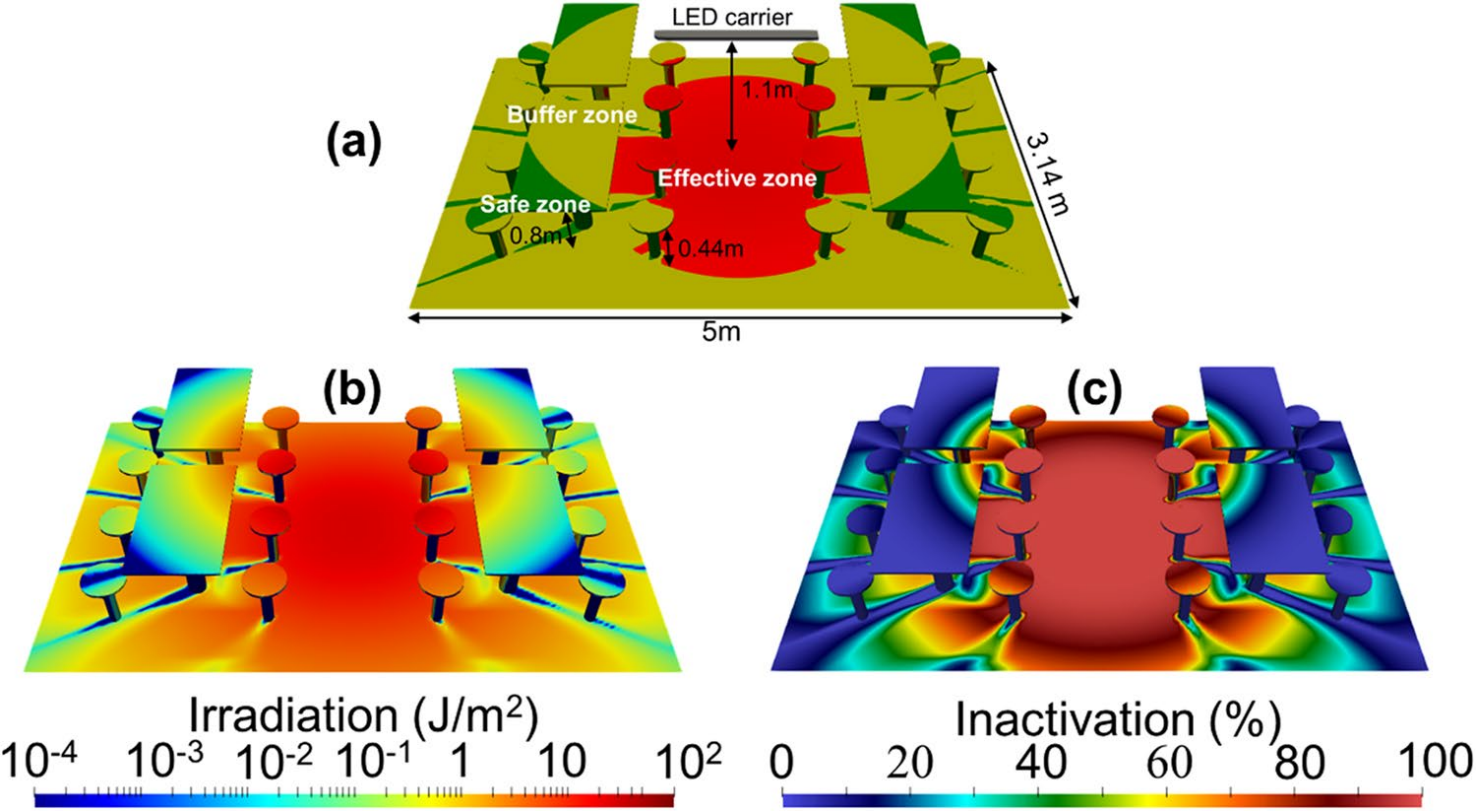
(**a**) The schematic feature of a typical food court in Singapore where the LED array (shown in Fig. 5a) is located in the center of the food court and 1.1-m from the ground. The zone map is defined (**b**) 3D irradiation mapping of the food court after a 30-s UV-C exposure, and the corresponding inactivation of SARS-CoV-2 evaluated from Eq. (3) is shown in (**c**).

Figure 6a shows a schematic of a typical table setting configuration in a public food court where the LED array is located in the center of the space with a distance of 1.1 m from the ground. The color mapping on the surface of the food court represents the zones at different UV-C irradiance levels as defined in Fig. 5a. According to the initial conditions, the simulated 3D UV-C irradiation mapping on the food court surface with a 30-s exposure time is shown in Fig. 6b. The value of irradiation is calculated from the time integration of emitted irradiance of the LED array, where the wavelength of the LED is 274 nm. The region near the LED array has higher UV-C irradiation. Otherwise, the regions far from the LED array, where the angle from the LED is large, and behind obstacles have a relatively low UV-C irradiation. Ignoring the radiative extinction due to the attenuation from the surface and the light path; *i.e.*, *η* = 0 and *α* = 0 in Eq. (4), the inactivation of the food court (see Fig. 6c) can be calculated using Eq. (3). Here the inactivation coefficient is set to 0.41, which is the value that corresponds to the wavelength of 274 nm (see Fig. 4). In addition, since the inactivation coefficient for the wavelength of 222 nm is also around 0.41, the activation map for 222 nm would be identical to that in Fig. 6c, assuming that the spatial radiative distribution of the LED's and arrangement of the LED array remain the same. Figure 6c shows that the distribution of inactivation is consistent with the zone map shown in Fig. 6a, as expected.

### Parametric analysis of the exposure time for sterilization

**Figure 7:**
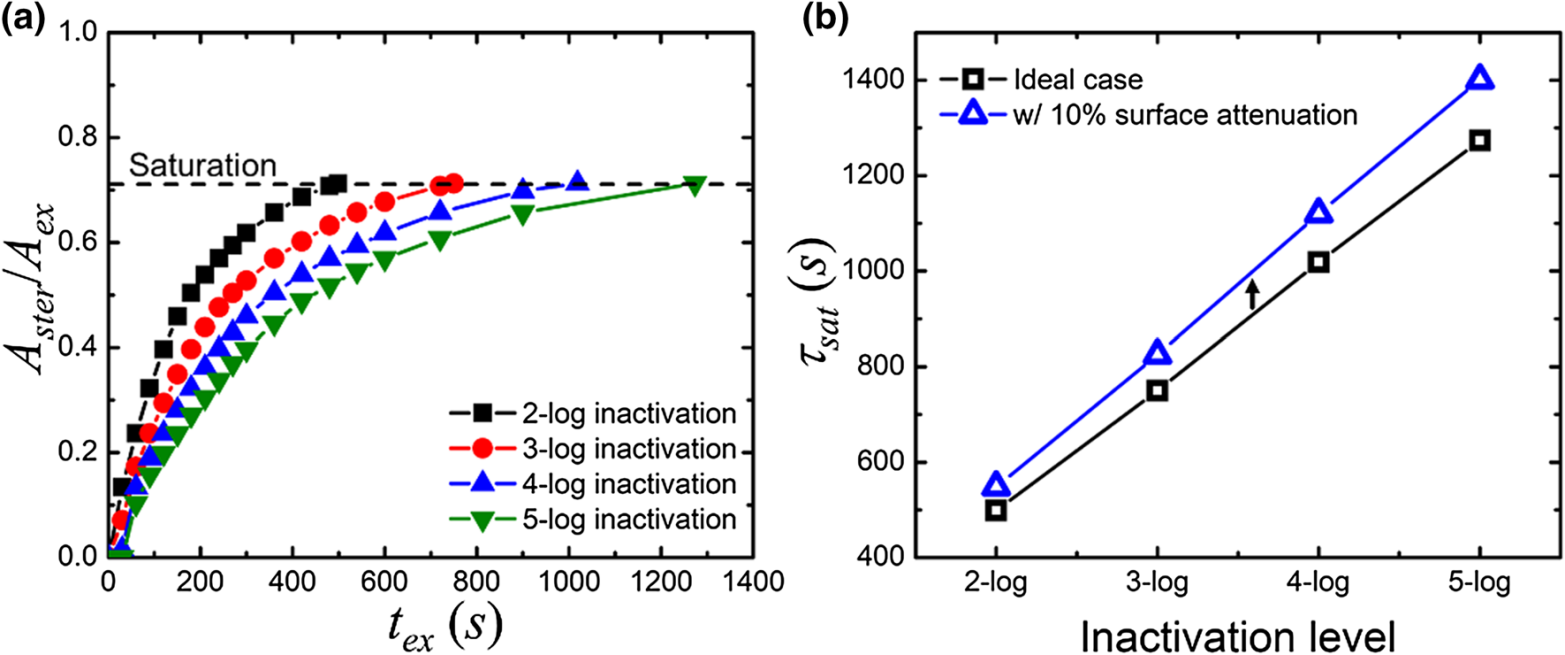
(**a**) Under different inactivation levels from 2-log to 5-log, *A*_ster_ /*A*_ex_ is a function of exposure time. *A*_ster_/*A*_ex_ increases and gradually saturates around 0.71 where the exposure time is defined as saturation time $\tau_{{{\text{sat}}}}$. (**b**) The effect of surface attenuation of UV-C radiation on the saturation time $\tau_{{{\text{sat}}}}$ is shown for inactivation levels from 2-log to 5-log.

To evaluate the exposure time for sterilization, Fig. 7a shows the ratio of the area *A*_ster_ with the specific disinfection level to the UV-C exposed area *A*_ex_ varying with the exposure time. The ratio parameter *A*_ster_/*A*_ex_ increases asymptotically with the exposure time *t*_*ex*_ from zero to a saturation point with an approximate value of 0.71. The saturation point is defined as the value of *A*_ster_/*A*_ex_ at which the rate of rising in *A*_ster_/*A*_ex_ is < 5% of the maximum rate of rising in the initial stage. The required time for the UV-C exposure to saturation is defined as $\tau_{{{\text{sat}}}}$. As the inactivation level increases, the LED array has a higher requirement of $\tau_{{{\text{sat}}}}$. For the usual viral inactivation (i.e., 3-log), $\tau_{{{\text{sat}}}}$ has to be > 10 min. $\tau_{{{\text{sat}}}}$ becomes > 20 min if the higher viral inactivation of 5-log is required. Figure 7b summarizes the values of $\tau_{{{\text{sat}}}}$ for different inactivation levels for the ideal case and when considering surface attenuation (i.e., *η* = 0.1). The surface attenuation of UV-C radiation increases the required $\tau_{{{\text{sat}}}}$ for all inactivation levels. The increment of $\tau_{{{\text{sat}}}}$ is proportional to the $\tau_{{{\text{sat}}}}$ itself for the specific inactivation level due to the linear approximation in Eq. (4).

**Figure 8:**
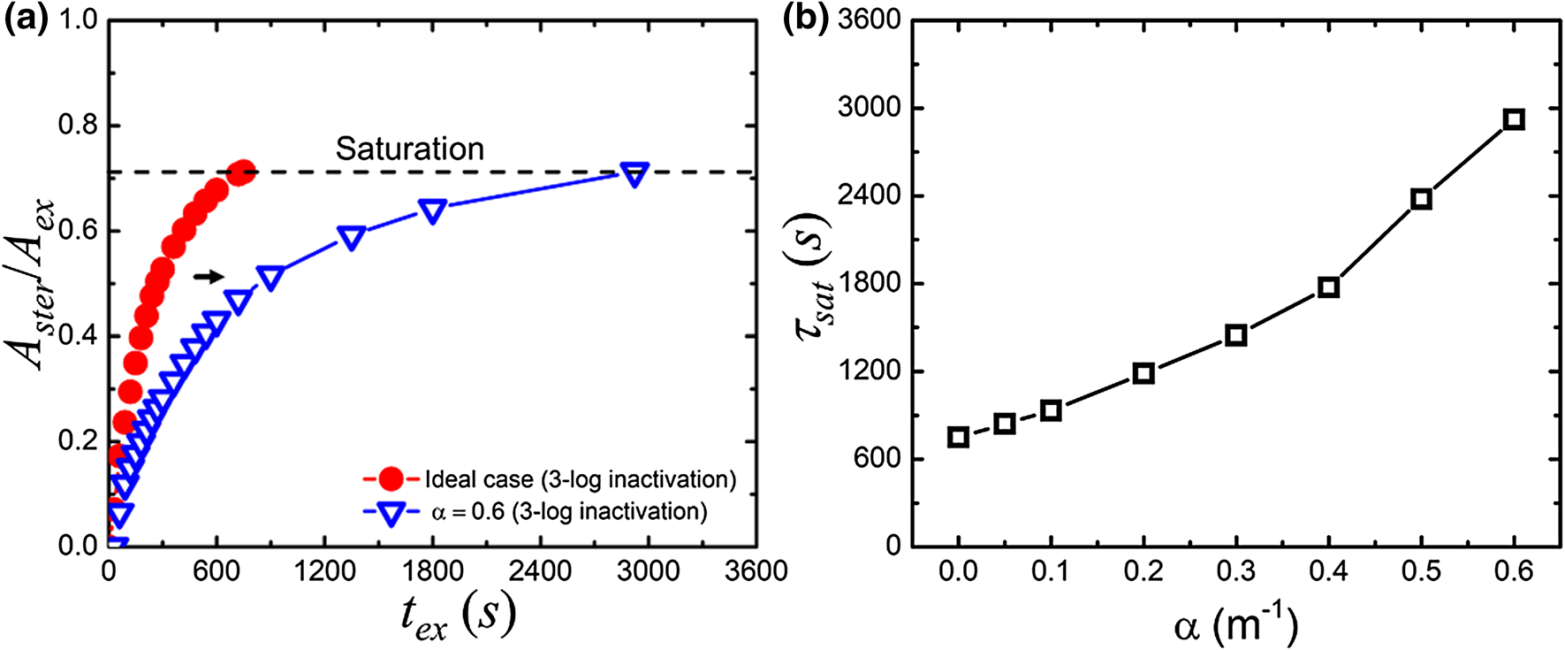
(**a**) The impact of extinction along the light path on the UV-C sterilization. (**b**) The saturation time $\tau_{{{\text{sat}}}}$ is a function of the extinction coefficient.

Each ray travels through intermediate substances in a straight path before it hits the target surface in the RT model. The substances may absorb and scatter the UV-C ray, and the radiant energy is attenuated along the light path. In this case, the extinction coefficient α may not be 0 in Eq. (4). Figure 8a shows the light extinction effect on the relationship between *A*_ster_/*A*_ex_ and the corresponding exposure time. Ideally, *A*_ster_/*A*_ex_ will attain saturation after 12.5-min of UV-C exposure. When α = 0.6, $\tau_{{{\text{sat}}}}$ will substantially increase to almost four times the original value. Figure 8b reveals the dependence of $\tau_{{{\text{sat}}}}$ versus α in which $\tau_{{{\text{sat}}}}$ increases significantly as α increases.

## Discussion

In Fig. 5a, the radiant characteristics of the specific-designed LED array are simulated using the RT model. We can evaluate the corresponding inactivation level from Eq. (3). According to the calculated UV-C intensity pattern in space, we divide the exposure region into the effective, buffer, and safe zones, respectively. To maximize the area for the effective UV-C germicide using the LED array, the intrinsic beam angle of a single LED is 120° and the distance to the ground is ~ 1.1 m, as revealed by Fig. 5b. It is worth noting that the optimised values of beam angle and distance to the ground apply to the specific design of the LED array, as presented in Fig. 5. In Fig. 6, we perform a case study of UV-C sterilization in a public environment using the RT model. In Fig. 6a, we differentiate the UV-C exposed surfaces of the food court into specific zones defined in Fig. 5a. From the simulated 3D irradiation in Fig. 6b and the calculated inactivation map in Fig. 6c, we define the parameter $\tau_{{{\text{sat}}}}$, which is the required time for the effective sterilization in the UV-C irradiation region. This parameter $\tau_{{{\text{sat}}}}$ is directly proportional to the UV-C dose for effective sterilization. In this way, adopting the UVC LEDs with higher operating power can reduce the required $\tau_{{{\text{sat}}}}$ proportionally to realize the efficient sterilization. As shown in Fig. 7a, the required $\tau_{{{\text{sat}}}}$ increases with the level of inactivation. The material property^19^, superficial structure^28^, and relative humidity^29^ of the target surface may degrade the magnitude of SARS-CoV-2 inactivation. The superficial dirt in the food court is commonly due to edible oil stains. For example, a layer of chicken oil with a thickness of 0.34 mm can cause 10% and 8.5% attenuation of UV-C irradiance in 222 nm and 274 nm, respectively^30^. In Fig. 7b, we evaluate $\tau_{{{\text{sat}}}}$ for the inactivation levels from 2-log to 5-log while assuming UV-C attenuation is 10% on the surface (i.e., *η* = 0.1). The increment of required $\tau_{{{\text{sat}}}}$ for the same inactivation level is simply proportional to $\tau_{{{\text{sat}}}}$ in the ideal case. If the UV-C light penetrates aerosols or encounters scattering and absorbing substances, we have to take into account the light extinction (i.e., *α* ≠ 0). For example, ozone has been commonly used for air disinfectants in which the concentration of ozone is even as high as 20 ppm^31^. In this case, the UV-C extinction due to ozone absorption at room temperature can be estimated and *α* is 0.6, 0.36, and 0.17 at the wavelength of 254, 274, and 222 nm, respectively^32^. Fig. 8a shows that $\tau_{{{\text{sat}}}}$ at *α* = 0.6 is 3.9 times the ideal case. From Fig. 8b, we can deduce that $\tau_{{{\text{sat}}}}$ required for 274 and 222-nm UV-C sterilization increases to 2.2 and 1.5 times when assuming 20-ppm ozone in the air.

## Conclusion

In conclusion, we have performed a feasibility study of UV-C LED for SARS-CoV-2 sterilization in the public environment using the race-tracing (RT) solver. The proposed in-house RT simulator can generate various irradiance maps of a typical UV-C LED point source with a beam angle of 40°, 120°, and 160°. By linear superposition of individual LEDs, it is possible to realize the specific design of the LED array. The benchmark simulation of a LED strip has been conducted, and the results from the RT solver have good agreements with those from experimental measurements, the commercial software (Zemax OpticStudio), and the analytical solution. To evaluate the SARS-CoV-2 inactivation quantitatively, the values of the inactivation coefficient (*K*) has been derived from the proposed experimental data^11,12^. According to the RT prediction of the irradiance profile of the emission from the germicidal system consisting of UV-C LEDs, we can clearly define the effective and safe zones in space and determine the optimised position to locate the UV-C germicidal system for the maximum area for the sterilization.

Moreover, a practical case study of a typical food court and the corresponding 3D UV-C irradiation mapping have been presented. From the predicted irradiation results, the viral inactivation map can be obtained. Based on the RT simulation results, the period ($\tau_{{{\text{sat}}}}$) of saturation of the UV-C exposure can be defined as the minimum required time for effective sterilization. $\tau_{{{\text{sat}}}}$ is directly proportional to the required UV-C dose and rises as the inactivation level increases. For further analysis of the impact of environmental factors on the SARS-CoV-2 inactivation, the superficial effect and the substance in the air should be considered as they could potentially degrade the SARS-CoV-2 inactivation. These physical mechanisms have been modeled in the RT solver and $\tau_{{{\text{sat}}}}$ becomes longer as compared with the ideal case. The proposed example shows that $\tau_{{{\text{sat}}}}$ is expected to be 2.2 and 1.5 times longer for UV-C sterilization at the wavelengths of 274 and 222-nm when assuming 20-ppm ozone in the air. To summarize, according to RT simulations, we provide a useful guideline to improve the safety and efficiency of usage of the UV-C-LED-based germicidal system. Furthermore, our simulation approach can numerically optimise other UV germicidal systems' designs in different environmental settings and extend the model for airborne/aerosol ultra-violet germicidal irradiation (UVGI) disinfection applications. Based on a physically accurate and geometrically flexible RT technique, the proposed simulator will be effectively applied in the light-based inactivation, including the novel light source (e.g., VUV^33^), on scenarios like aircraft cabins, hospitals, and shopping malls. In this case, the accurate experimental measurement of the corresponding environmental parameters *η*, *α*, and *K* versus the operating wavelength of the light source remains to be done.
